# Association between acute gastrointestinal injury and biomarkers of intestinal barrier function in critically ill patients

**DOI:** 10.1186/s12876-017-0603-z

**Published:** 2017-03-29

**Authors:** Hongxiang Li, Ying Chen, Feifei Huo, Yushan Wang, Dong Zhang

**Affiliations:** grid.430605.4 ICU, First Hospital of Jilin University, No. 71 Xinmin Street, Changchun, 130021 China

**Keywords:** Acute gastrointestinal injury, Grade, Intestinal barrier function, Hyperpermeability, Biomarker

## Abstract

**Background:**

To assess the associations of biomarkers of intestinal barrier function and other clinical variables with acute gastrointestinal injury (AGI) grade, and of these clinical variables with mortality in critically ill patients.

**Methods:**

This was a single-center, observational, prospective study. Patients were included if they were diagnosed with AGI and underwent tests for the measurement of plasma levels of intestinal fatty acid–binding protein (i-FABP), d-lactate (d-la), and lipopolysaccharide. General characteristics, AGI grades, Acute Physiology and Chronic Health Evaluation (APACHE) II scores, Sepsis-related Organ Failure Assessment (SOFA) scores, intra-abdominal pressure (IAP), and 28-day mortality were recorded and compared among patients with different AGI grades.

**Results:**

Among the 90 included patients, the APACHE II score, IAP, and LPS and D-la levels significantly differed between the four AGI grades. Multinomial logistic regression analysis with grade I as the reference for grades II, III, and IV revealed that high APACHE II scores increased the odds of AGI grade III (odds ratio [OR], 1.754; 95% confidence interval [CI], 1.225–2.511) and grade IV (OR, 1.493; 95% CI, 1.079–2.066). Similarly, IAP increased the odds of AGI grade III (OR, 1.622; 95% CI, 1.111–2.369) and grade IV (OR, 1.518; 95% CI, 1.066–2.162). Elevated D-la increased the odds of AGI grades II (OR, 1.059; 95% CI, 1.005–1.117), III (OR, 1.155; 95% CI, 1.052–2.268), and IV (OR, 1.088; 95% CI, 1.013–1.168). In contrast, i-FABP and LPS did not increase the odds of any AGI grade. SOFA scores could independently predict the odds of death in AGI patients (OR, 1.223; 95% CI, 1.007–1.485).

**Conclusion:**

AGI patients exhibit loss of gastrointestinal barrier function, and d-la could serve as a better marker of AGI grade than i-FABP or lipopolysaccharide.

## Background

In critically ill patients, the intestine is a vulnerable organ, and gastrointestinal (GI) dysfunction is common [1]. Conversely, GI dysfunction can indicate a critical condition. It has been reported that almost 50% of patients in intensive care units (ICUs) have enterocyte damage at admission [2]. Among critically ill patients, those with GI dysfunction have higher mortality rates than those without GI dysfunction. [3, 4]. It is therefore important to monitor the status of the GI tract in critically ill patients.

In 2012, the Working Group on Abdominal Problems of the European Society of Intensive Care Medicine (ESICM) defined acute gastrointestinal injury (AGI) as the malfunctioning of the GI tract in critically ill patients due to their acute illness, and recommended a four-grade classification for AGI severity [5]. However, this definition mainly depends on the symptoms and signs of AGI, which are usually not sufficient to diagnose the underlying disease [6]. Some biomarkers, for example, blood intestinal fatty acid–binding protein (i-FABP), d-lactate (d-la), and lipopolysaccharide (LPS), have been proposed as possible markers for intestinal barrier function and the detection of AGI [7]. However, their clinical validity in the diagnosis and classification of AGI is still unclear. To our knowledge, no study has evaluated these biomarkers in critically ill patients with varying grades of AGI severity.

The purpose of this study was to determine whether biomarkers of GI barrier function could be used to indicate the severity and prognosis of AGI in critically ill patients. To this end, we assessed the association of various clinical parameters and biomarkers of GI barrier function with AGI severity and 28-day mortality in critically ill patients.

## Methods

### Study design

This prospective, observational study was conducted to assess the correlation of certain biomarkers in critically ill patients with different AGI grades. This study was performed in a 25-bed general ICU at the First Hospital of Jilin University (Changchun, China) from January 1, 2014 to June 30, 2014.

### Patient selection and grouping

Patients were included if they had been hospitalized for at least 72 h before being diagnosed with AGI, according to the ESICM definition [5]. Patients were excluded from the study if they were less than 18 years old; diagnosed with a malignancy, Crohn disease, ulcerative colitis, or short bowel syndrome; or hospitalized for less than 72 h before the AGI diagnosis was established.

The patients were divided into four groups based on the ESICM-recommended four-grade classification (grades I, II, III, and IV), which in turn is based on the calorie amount of enteral nutrition and intra-abdominal pressure (IAP) (Table 1) [5].

**Table 1:** Classification of AGI [5]

Grade	Definition
I (risk of GI dysfunction or failure)	Partial impairment of GI function, manifested as gastrointestinal symptoms related to a known cause and perceived to be transient. Examples: postoperative nausea and/or vomiting during the first few days after abdominal surgery, postoperative absence of bowel sounds, diminished bowel motility in the early phase of shock.
II (GI dysfunction)	The GI tract is unable to perform digestion and absorption adequately to satisfy the nutrient and fluid requirements of the body. There are no changes in the general condition of the patient due to GI problems. Examples: gastroparesis with high gastric residuals or reflux, paralysis of the lower GI tract, diarrhea, intra-abdominal pressure (IAP) 12–15 mmHg, visible blood in gastric content or stool. Feeding intolerance is present if at least 20 kcal/kg BW/day via the enteral route cannot be achieved within 72 h of a feeding attempt.
III (GI failure)	Loss of GI function. Restoration of GI function is not achieved despite interventions, and the general condition is not improving. Examples: persistent feeding intolerance despite treatment manifested as high gastric residuals, persistent GI paralysis, occurrence or worsening of bowel dilatation, IAP, 15–20 mmHg, low abdominal perfusion pressure (below 60 mmHg). Feeding intolerance is present and possibly associated with persistence or worsening of multiple organ dysfunction syndrome.
IV (GI failure with severe impact on distant organ function)	AGI has progressed to become directly and immediately life-threatening, with worsening of multiple organ dysfunction syndrome and shock. Examples: bowel ischemia with necrosis, GI bleeding leading to hemorrhagic shock, Ogilvie syndrome, abdominal compartment syndrome requiring decompression.

Primary AGI is associated with primary disease or direct injury to organs of the GI system, such as peritonitis, pancreatitis, abdominal surgery. Secondary AGI develops as a consequence of the host response to critical illness without a primary pathology in the GI system, such as GI malfunction in a patient with pneumonia or non-abdominal surgery

*AGI* acute gastrointestinal injury, *BW* body weight, *GI* gastrointestinal, *IAP* intra-abdominal pressure

### Data collection and clinical evaluation

Nutritional support and other treatments were provided according to local practice guidelines and the clinicians’ discretion. Blood samples to detect GI injury were collected on the day the patient was diagnosed with AGI. The samples were centrifuged, and the plasma thus obtained was frozen at −20 °C and sent to the laboratory within 1 week for analysis. Plasma i-FABP, d-la, and LPS levels were measured using enzyme-linked immunosorbent assay kits (R&D Systems, Minneapolis, USA). We recruited 50 healthy volunteers and measured their plasma i-FABP, d-la, and LPS levels as a reference.

The following data were acquired from the patients: general characteristics, AGI grade, IAP (highest value obtained on bladder manometry in the first 3 days, with each measurement being performed at a set time of the day; measurements were performed at least 4 times a day, if the IAP exceeded 12 mm Hg, and mean values were used [3]), abdominal perfusion pressure (APP; difference between mean blood pressure and IAP, determined at the time of IAP measurement), Acute Physiology and Chronic Health Evaluation (APACHE) II score (in the first 24 h after ICU admission), Sepsis-related Organ Failure Assessment (SOFA) score (in the first 24 h after ICU admission), and 28-day mortality.

### Statistical analyses

Categorical variables are presented as percentages, whereas continuous variables are presented as median and interquartile range (IQR). Variables involved in the four-grade AGI classification were compared using the Kruskal–Wallis test. Variables that were statistically significant (*p* < 0.05) were included in the multinomial multiple logistic regression analysis (method: enter) to identify associations between AGI grade and specific parameters. These variables were also included in the ordinal logistic regression analysis with AGI grades as the dependent variable to identify associations between ranked AGI grade and specific parameters. The associations between APACHE II score, SOFA score, AGI grade, IAP, APP, mortality, and LPS, d-la, and i-FABP levels were assessed using univariate analysis and multiple logistic regression analysis (method: enter).

A *p*-value of <0.05 was considered statistically significant. All tests were two-sided. Data were analyzed using commercially available software (PASW Statistics, version 17.0; SPSS, Chicago, IL, USA).

## Results

### Patient enrollment

**Fig. 1:**
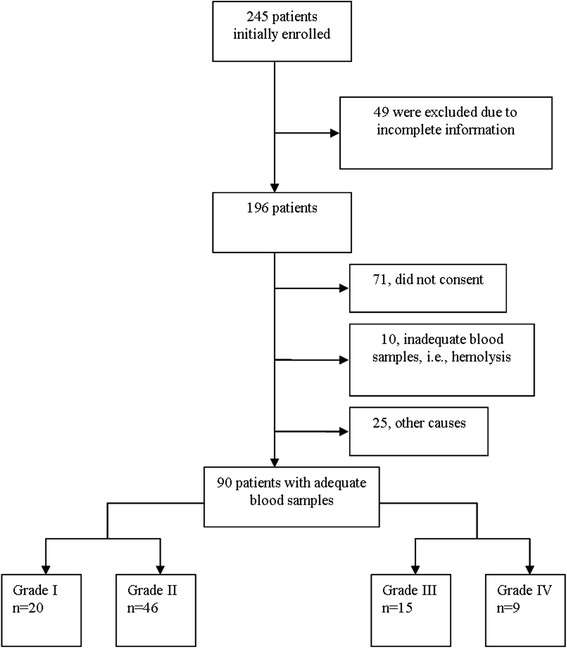
Flow chart of patient selection

Of the 245 patients initially enrolled in the study, 49 were excluded due to lack of complete information, loss to follow-up, or an unclear AGI classification; 71 patients did not provide consent; 10 had inadequate blood samples; and 25 were excluded due to other reasons such as problems with operational approaches and safekeeping. Thus, a total of 90 patients (grade I, 20 patients; grade II, 46 patients; grade III, 15 patients, and grade IV, 9 patients) were included in the analysis (Fig. 1).

### Baseline characteristics of the patients

Among the 90 included patients, the median age was 66 years (IQR, 47.0–80.0 years), the median APACHE II score was 20.0 (IQR, 16.0–22.0), the median SOFA score was 6.0 (IQR, 4.0–9.0), and the 28-d mortality was 14 (15.6%; Table 2).

**Table 2:** Baseline characteristics of patients with AGI

Variable	I *n* = 20	II *n* = 46	III *n* = 15	IV *n* = 9	Total *n* = 90	*P*
Age (yr)	66 (48–78)	68 (47–82)	74 (43–87)	69 (61–81)	66.0 (47.0–80.0)	0.959
Males	14 (70%)	34 (73.9%)	14 (93.3%)	7 (77.8%)	69 (76.7%)	0.393
APACHE II score	20.0 (16.5–22.0)	18.0 (11.8–22.0)	20.0 (18.0–30.0)	23.0 (18.0–28.5)	20.0 (16.0–22.0)	0.031^a^
SOFA score	6.0 (3.0–8.0)	5.0 (4.0–9.0)	6.0 (4.0–13.0)	8.0 (4.5–11.5)	6.0 (4.0–9.0)	0.486
Gastrointestinal surgery	1 (20%)	5 (10.9%)	1 (6.7%)	1 (11.1%)	8 (8.9%)	0.262
Primary AGI	3 (15%)	17 (37%)	5 (33.3%)	5 (55.6%)	30 (33.3%)	0.155
Sepsis	8 (40%)	19 (41.3%)	4 (26.7%)	4 (44.4%)	35 (38.9%)	0.760
Catecholamine support	5 (25%)	8 (17.4%)	5 (33.3%)	5 (55.6%)	23 (25.6%)	0.099
Mechanical ventilation	17 (85%)	34 (73.9%)	11 (73.3%)	9 (100%)	71 (78.9%)	0.281
CRRT	2 (10%)	10 (21.7%)	1 (6.7%)	1 (11.1%)	14 (15.6%)	0.421
Primary reason for intensive care						0.131
Acute pancreatitis	1 (5%)	13 (28.3%)	5 (33.3%)	1 (11.1%)	20 (22.2%)	
Shock	3 (15%)	8 (17.4%)	1 (6.7%)	3 (33.3%)	15 (16.6%)	
AKI	2 (10%)	10 (21.7%)	1 (6.7%)	4 (44.4%)	17 (18.9%)	
ARDS	8 (40%)	14 (30.4%)	6 (40%)	5 (55.6%)	33 (36.7%)	
Trauma	2 (10%)	6 (13%)	0	0	8 (8.9%)	
Postoperative	3 (15%)	2 (4.3%)	2 (13.3%)	0	7 (7.8%)	
Cardiac arrest	1 (5%)	1 (2.2%)	1 (6.7%)	0	3 (3.3%)	
28 d-mortality	1 (5.0%)	7 (15.2%)	2 (13.3%)	4 (44.4%)	14 (15.6%)	0.067

Measurement values are expressed as median (interquartile range, 25%–75%). Categorical variables are reported as n (%). Variables were compared using the Kruskal–Wallis test

*AGI* acute gastrointestinal injury, *ARDS* acute respiratory distress syndrome, *APACHE* acute physiology and chronic health evaluation, *AKI* acute kidney injury, *CRRT* continuous renal replacement therapy, *SOFA* sepsis-related organ failure assessment

^a^Significant differences among AGI grades I, II, III, and IV

### Association between AGI grade and patient characteristics

The median IAP and APP values were 13.0 mm Hg (IQR, 9.0–14.0 mm Hg) and 77.0 mm Hg (IQR, 63.0–90.0 mm Hg), respectively. The median plasma i-FABP, LPS, and d-la concentrations were 551.6 pg/mL (IQR, 438.9–660.0 pg/mL), 5.9 pg/mL (IQR, 3.9–7.7 pg/mL), and 31.2 μmol/L (IQR, 16.1–59.7 μmol/L), respectively (Table 3). All of these concentrations were higher than the corresponding reference values (Table 4).

**Table 3:** Laboratory data and other variables of patients with AGI

Variable	I *n* = 20	II *n* = 46	III *n* = 15	IV *n* = 9	Total *n* = 90	*P*
Serum albumin (g/L)	27.1 (22.9–31.2)	31.6 (26.4–34.6)	29.8 (26.6–34.2)	28.3 (20.7–29.7)	30.0 (24.2–32.9)	0.077
CRP (mg/L)	141.0 (115.5–165.1)	109.6 (41.8–195.0)	96.7 (23.4–171.7)	96.6 (61.0–189.2)	116.3 (48.6–188.0)	0.781
PCT (μg/L)	3.9 (0.8–8.5)	1.7 (0.2–17.6)	4.8 (0.9–6.0)	11.3 (4.3–27.1)	2.7 (0.4–11.3)	0.580
Arterial lactate (mmol/L)	1.4 (1.1–2.0)	1.7 (1.1–3.3)	1.9 (1.1–2.7)	2.7 (1.5–3.9)	1.7 (1.2–2.7)	0.167
IAP (mm Hg)	11.0 (8.5–13.0)	13.0 (8.5–14.0)	14.5 (10.3–17.50)	14.0 (11.5–18.5)	13.0 (9.0–14.0)	0.042^a^
APP (mm Hg)	82.0 (66.5–92.0)	77.0 (70.5–91.0)	79.5 (58.0–88.3)	69.0 (44.0–87.5)	77.0 (63.0–90.0)	0.639
i-FABP (pg/mL)	516.1 (422.0–662.6)	518.0 (423.2–622.2)	597.4 (488.2–657.0)	752.3 (540.9–3208.9)	551.6 (438.9–660.0)	0.051^a^
LPS (pg/mL)	4.6 (3.4–7.4)	5.5 (3.7–7.7)	6.2 (5.0–7.3)	9.0 (7.1–45.5)	5.9 (3.9–7.7)	0.008^a^
d-la (μmol/L)	16.8 (14.0–44.6)	28.0 (17.0–60.5)	58.9 (20.7–62.9)	52.1 (33.4–223.0)	31.2 (16.1–59.7)	0.012^a^

Measurement values are expressed as median (interquartile range, 25%–75%). Categorical variables are reported as n (%). Variables were compared using the Kruskal–Wallis test

*AGI* acute gastrointestinal injury, *APP* abdominal perfusion pressure, *CRP* C-reactive protein, *d* *-la* d-lactate, *IAP* intra-abdominal pressure, *i-FABP* intestinal fatty acid–binding protein, *LPS* lipopolysaccharide, *PCT* procalcitonin

^a^Significant differences among AGI grades I, II, III, and IV

**Table 4:** Reference values of plasma i-FABP, LPS, and d-la in healthy individuals

Biomarker	Median (IQR, 25%–75%)
i-FABP (pg/mL)	31.32 (24.54–34.87)
LPS (pg/mL)	2.65 (1.17–3.45)
d-la (μmol/L)	8.21 (3.23–10.37)

Values were obtained from 50 volunteers (men, 40%) with a mean age of 47 ± 15 years. The volunteers were recruited from communities in Changchun, and were adults without any history of malignancy, infections, or gastrointestinal disease in the last 3 months

*d* *-la* d-lactate, *i-FABP* intestinal fatty acid–binding protein, *IQR* interquartile range, *LPS* lipopolysaccharide

The APACHE II score, IAP, and LPS and D-la levels significantly differed among patients with grade I, II, III, and IV AGI (Tables 2 and 3). Multinomial logistic regression analysis with grade I as the reference for grades II, III, and IV revealed that high APACHE II score increased the odds of AGI grades III (odds ratio [OR], 1.754; 95% confidence interval [CI], 1.225–2.511) and IV (OR, 1.493; 95% CI, 1.079–2.066). Similarly, IAP increased the odds of grade III (OR, 1.622; 95% CI, 1.111–2.369) and grade IV AGI (OR, 1.518; 95% CI, 1.066–2.162). Elevated D-la increased the odds of AGI grade II (OR, 1.059; 95% CI, 1.005–1.117), grade III (OR, 1.155; 95% CI, 1.052–2.268), and grade IV (OR, 1.088; 95% CI, 1.013–1.168). In contrast, i-FABP and LPS did not increase the odds of any AGI grade (Table 5). Ordinal logistic regression analysis revealed that APACHE II score (OR, 1.115; 95% CI, 1.106–1.222), IAP (OR, 1.143; 95% CI, 1.031–2.267), and D-la (OR, 1.043; 95% CI, 1.013–1.074) were independently associated with ranked AGI grades (Table 6).

**Table 5:** Multiple regression analysis of characteristics of the patients divided by AGI grade

	Grade II		Grade III		Grade IV	
	OR (95% CI)	*P*	OR (95% CI)	*P*	OR (95% CI)	*P*
APACHE II score	1.017 (0.884–1.168)	0.817	1.754 (1.225–2.511)	0.002	1.493 (1.079–2.066)	0.015
IAP (mm Hg)	1.174 (0.890–1.550)	0.257	1.622 (1.111–2.369)	0.012	1.518 (1.066–2.162)	0.021
i-FABP (pg/mL)	0.993 (0.987–1.000)	0. 067	0.996 (0.985–1.007)	0. 454	0.997 (0.987–1.007)	0.575
LPS (pg/mL)	1.286 (0.846–1.956)	0.239	0.609 (0.238–1.559)	0.301	0.923 (0.497–1.714)	0.800
d-la (μmol/L)	1.059 (1.005–1.117)	0.033	1.155 (1.052–1.268)	0.003	1.088 (1.013–1.168)	0.021

Variables were compared using multinomial logistic regression for the multiple analysis; Grade I is the reference for Grades II, III, and IV

*AGI* acute gastrointestinal injury, *APACHE* acute physiology and chronic health evaluation, *CI* confidence interval, *d* *-la* d-lactate, *IAP* intra-abdominal pressure, *i-FABP* intestinal fatty acid–binding protein, *LPS* lipopolysaccharide, *OR* odds ratio

**Table 6:** Ordinal logistic regression analysis of variables to predict AGI grade

	OR (95% CI)	*P*
APACHE II score	1.115 (1.106–1.222)	0.021
IAP (mm Hg)	1.143 (1.031–1.267)	0.011
LPS (pg/mL)	1.077 (0.913–1.271)	0.377
d-la (μmol/L)	1.043 (1.013–1.074)	0.004
i-FABP (pg/mL)	0.997 (0.993–1.000)	0.053

*AGI* acute gastrointestinal injury, *APACHE* acute physiology and chronic health evaluation, *CI* confidence interval, *d* *-la* d-lactate, *IAP* intra-abdominal pressure, *i-FABP* intestinal fatty acid–binding protein, *LPS* lipopolysaccharide, *OR* odds ratio

### Association of AGI-related clinical variables with 28-day mortality

A regression analysis was conducted to determine the association of various clinical variables with 28-d mortality (Table 7). Univariate regression analysis showed that higher APACHE II score, SOFA score, and AGI grade significantly increased the odds of death in AGI patients. In the multiple regression analysis, however, only the SOFA score could independently predict the odds of death in AGI patients (OR, 1.223; 95% CI, 1.007–1.485).

**Table 7:** Regression analysis of variables to predict 28-day mortality

	Univariate analysis		Multiple analysis	
	OR (95% CI)	*P*	OR (95% CI)	*p*
APACHE II score	1.120 (1.021–1.229)	0.017	1.042 (0.862–1.260)	0.672
SOFA score	1.240 (1.081–1.422)	0.002	1.223 (1.007–1.485)	0.042
AGI grade	2.042 (1.007–3.873)	0.029	1.658 (0.616–4.463)	0.317
IAP (mm Hg)	0.975 (0.858–1.109)	0.701	0.887 (0.726–1.084)	0.243
LPS (pg/mL)	1.016 (0.991–1.041)	0.207	0.953 (0.796–1.140)	0.598
d-la (μmol/L)	1.005 (0.999–1.012)	0.080	1.011 (0.979–1.044)	0.495
i-FABP (pg/mL)	1.000 (1.000–1.001)	0.147	1.000 (0.997–1.004)	0.944

Variables were compared using binary logistic regression for the multiple analysis; survival group is the reference for death group

*AGI* acute gastrointestinal injury, *APACHE* acute physiology and chronic health evaluation, *APP* abdominal perfusion pressure, *CI* confidence interval; d-la, d-lactate, *IAP* intra-abdominal pressure, *i-FABP* intestinal fatty acid–binding protein, *LPS* lipopolysaccharide, *OR* odds ratio, *SOFA* sepsis-related organ failure assessment

## Discussion

This study analyzed the association of certain markers of GI barrier function with AGI severity and mortality in critically ill patients. The results showed that the APACHE II score, IAP, and d-la level could reflect AGI severity, while only the SOFA score could independently predict the odds of death in AGI patients.

GI functions include the absorption of nutrients and water, barrier control to modulate absorption of intraluminal microbes (and their products), and endocrine and immune functions [5]. However, we currently lack the tools or markers to comprehensively measure GI function, and thus, we cannot reliably evaluate this in the acute setting. Although the Recommendations of the ESICM Working Group on Abdominal Problems defined AGI and its grading, this definition is mainly based on the functions of digestion and absorption, and does not reflect other GI functions, such as acting as a barrier to harmful intraluminal substances [5].

Studies have indicated that the development of multiple organ dysfunction syndrome (MODS) is associated with a derangement in intestinal permeability that is detectable before the onset of the MODS [8]. Meakins and Marchall postulated that the gut serves as the “motor” of MODS in injured or critically ill patients [9]. Dysfunction of the GI barrier mainly manifests as intestinal epithelial hyperpermeability [10], which is common during critical illness. Therefore, it is reasonable to suggest that alteration of the GI barrier function plays an important role in the development MODS or GI injury, and should be taken into account during AGI grading.

The intestinal barrier is a protective component of the gut, shielding us from bacterial invasion or invasion by other microorganisms and their toxins. Intestinal permeability can be understood as a measurable feature of the intestinal barrier. Barrier function may be quantified by measuring the translocation of bacteria or bacterial products, such as LPS and d-la, from the gut into the portal vein or the systemic circulation or by assaying biomarkers of epithelial cell integrity such as i-FABP. Few studies have investigated the role of intestinal barrier function in AGI grading. We selected three biomarkers—LPS, d-la, and i-FABP—that reflect intestinal permeability to evaluate intestinal barrier function [10].

LPS is a glycolipid present in the outer membrane of gram-negative bacterial cell walls [11]. The mucosal epithelium of the gastrointestinal tract serves as a major barrier to LPS, whereas the bacteria present in the intestinal lumen act as a major source of LPS [12, 13]. The process of LPS translocation from the gastrointestinal lumen to the systemic circulation is not fully understood, but it is thought that the GI tract is rendered permeable to LPS through changes in tight junctions [14]. Hence, an increased serum level of LPS can reflect GI barrier dysfunction and increased intestinal permeability. This study found that the LPS level could distinguish between the different AGI grades on univariate analysis, but not on multiple regression analysis, possibly due to the influence of sepsis. Increased peripheral blood LPS levels have been detected in sepsis [15], and in the present study, a high proportion (about 40%) of the enrolled patients had sepsis, which could have interfered in the relationship between blood LPS levels and AGI grade.

Fatty acid–binding proteins are small cytosolic water-soluble proteins present in mature enterocytes. The levels of i-FABP have been reported to reflect the physiological turnover rate of enterocytes, with elevated levels indicating intestinal epithelial cell damage [16]. Thus, the i-FABP level could be a useful marker for the early detection of significant intestinal injury [17]. The i-FABP level has been found to correlate with the gut dysfunction score in acute pancreatitis [18]. The present study found that i-FABP could distinguish between different AGI grades on univariate analysis, but not on multiple regression analysis, which included other factors that distinguished between AGI grades.

Normally, lactic acid exists in the form of l-lactate in mammalian cells, which almost exclusively produce this form of lactic acid. d-la is a fermentation product generated by many bacteria, including those present in the human GI tract [19]. Low circulating levels of d-la are found in healthy individuals, but in the event of intestinal barrier function loss, these levels will rise as a consequence of increased translocation across the intestinal mucosa [20]. An increase in plasma d-la has been associated with intestinal ischemia [21]. Elevated serum d-la levels have been recorded in animals with high IAP, and a positive correlation was detected between blood d-la levels and IAP, and blood d-la levels may be an early indicator of increased IAP before intestinal ischemic changes occur [22]. A relationship between plasma d-la and colonic permeability has been suggested [23]. However, research on d-la and GI barrier function has not yet conclusively proven the value of this marker in evaluating AGI. Moreover, increases in plasma d-la levels have been detected in other conditions with non- impaired intestinal barrier, such as short-bowel syndrome, due to excessive GI fermentation of carbohydrates [24]. However, these conditions also affect GI dysfunction. Hence, we consider that d-la is a good potential marker of GI injury that may influence the choices of AGI therapy in critically ill patients, and thus merits further study.

We also evaluated some clinical parameters associated AGI, such as AGI type (primary or secondary), GI surgery, serum albumin, sepsis, serum C-reactive protein, serum procalcitonin, arterial lactate, catecholamine support, SOFA score, APACHE II score, and IAP. We found APACHE II score and IAP could distinguish between the different AGI grades on multiple regression analysis. The finding that APACHE II scores could distinguish between different AGI grades proves the rationality of the AGI classification [6]. Although the value of IAP in AGI evaluation is controversial, IAP is an important factor determining GI failure [3] and ESICM-defined AGI. Thus, it is reasonable that IAP could distinguish between different AGI grades.

The SOFA score, a valuable organ injury score that has been proven to predict prognosis in critical ill patients [25], is not based on AGI biomarkers. Thus, it may be worthwhile to determine the association of AGI biomarkers with SOFA scores in future studies. In this study, we found that blood d-la levels could distinguish AGI grades on both univariate analysis and multiple regression analysis, proving that the plasma d-la level can be a better biomarker of AGI grade than i-FABP and LPS levels. Thus, plasma d-la levels may be helpful to distinguish the severity of AGI and to monitor the progression of AGI. The usefulness of this biomarker in guiding treatment decisions and assessing therapeutic outcomes should be assessed in future studies.

Our study has certain limitations. First, the sample size was relatively small, and it is difficult to exclude the effects of confounding factors due to the diverse baseline characteristics of critically ill patients. Second, there was no control group of critically ill patients with normal GI function. Third, i-FABP, LPS, and d-la only partially reflect intestinal barrier function, and tight junction molecules such as claudins and zonula occludens [26], were not detected in this study. Fourth, methods of nutritional support were not included in the analysis.

## Conclusions

AGI patients exhibit injury to the gastrointestinal barrier, and d-la could serve as a better marker of AGI grade than i-FABP or LPS. In addition to d-la, APACHE II score and IAP were associated with AGI grade in this study. The SOFA score was confirmed to be useful in predicting the prognosis of critically ill patients with AGI.

## Abbreviations

AGI: Acute gastrointestinal injury
APACHE: Acute physiology and chronic health evaluation
APP: Abdominal perfusion pressure
d-la: d-lactate
ESICM: European Society of Intensive Care Medicine
GI: Gastrointestinal
IAP: Intra-abdominal pressure
ICU: Intensive care unit
i-FABP: Intestinal fatty acid–binding protein
IQR: Interquartile range
LPS: Lipopolysaccharide
MODS: Multiple organ dysfunction syndrome
SOFA: Sepsis-related organ failure assessment
